# The mediating effect of cognitive appraisal on the relationship between sleep habits and the stress response among Japanese female college students

**DOI:** 10.1186/s40359-021-00602-w

**Published:** 2021-06-26

**Authors:** Noriko Aizawa, Mika Omori

**Affiliations:** 1 Institute for Education and Human Development, Ochanomizu University, 2-1-1 Bunkyo-ku, Otsuka, Tokyo, Japan; 2 Department of Psychology, Ochanomizu University, Tokyo, Japan; 3 Department of Psychology, Tohoku University, Sendai, Japan

**Keywords:** Cognitive appraisal, Female college students, Sleep habits, Sleep irregularity, Sleep pattern, Stress response

## Abstract

**Background:**

Undergraduate students tend to develop “evening-type” sleep patterns. Recent research has reported that evening-type and irregular sleep habits are related to physical and mental stress responses, particularly in female students. Although the connection between sleep habits and the stress response has been well documented, the mechanism behind this relationship is currently unknown. Using the transactional model as a framework and female students as our target population, we examined whether sleep habits predict the stress response through the mediation of cognitive appraisals of one’s own sleep habits.

**Methods:**

Three hundred twenty-one Japanese female college students participated in this study. Participants completed measures of their sleep habits (sleep patterns and sleep irregularity), cognitive appraisals of their sleep habits (including four subscales: commitment, appraisal of influence, appraisal of threat, and controllability), stress responses (depression and anxiety), and control factors. The reliability and validity of the scales used in this study, except for sleep pattern and sleep irregularity, were confirmed in previous studies.

**Results:**

Multiple-mediation-model analysis indicated that commitment mediates the relationship between sleep pattern and anxiety. Meanwhile, cognitive appraisals, as a whole, were found to have a mediating effect on the relationship between sleep irregularity and depression.

**Conclusions:**

Our study revealed that cognitive appraisals mediated the relationship between sleep habits and the stress response. The findings also suggest that maintaining a low level of commitment might be effective for reducing anxiety, especially considering the difficulty associated with changing lifestyles. The findings of the present study should be useful for health education related to lifestyle.

## Background

In recent years, an increasing number of undergraduate students have begun to exhibit “evening-type” rhythms in their daily lives [1–4]. This refers to people who are more active from evening till night. In Japan, approximately 50% of the 18-year-old population is enrolled in colleges or universities [5]. The primary reason that these students become evening-type and develop irregular sleep habits is related to the freedom associated with college life, such as reductions in interference and constraints from their families and school and more liberty to organize their schedules [1, 6]. One study of college students found that the average bedtime was 1:15 a.m. [2], with 74% of female students reporting going to bed between 12:00 a.m. and 2:30 a.m. [7]. Based on these findings, it appears that most university students commence their sleeping phase post-midnight, despite midnight being the most common bedtime for the general adult population in Japan [8]. Thus, the evening-type lifestyle seems to be a characteristic of Japanese undergraduate students [3, 4].

Several studies have reported that evening-types exhibit an efficacious stress response than morning types [9]. It has also been reported that evening-type sleeping habits were related to poor physical and mental health conditions [10–14]. For example, studies of undergraduate students have established that evening-types had a less efficient autonomic nervous system functioning in the morning [12], higher levels of psychological stress response [11], poorer physical and mental health [10], and lower academic performance [15] than morning types. Matsui et al. [16] found that university students with evening-type lifestyles reported relatively more fatigue and feelings of sickness, while Honda et al. [17] found that students with morning-type lifestyles showed a lower rate of such indefinite complaints. Evidently, sleep is also an important factor and has been reported to affect mental stress responses. For example, poor sleep quality has been associated with depression and anxiety in adults and college students in North and South America [18–20]. Thus, based on existing findings, it is evident that sleep habits are associated with negative psychological and physical stress responses.

The impact of sleeping habits on the mind and body may be an especially important issue for female college students. For example, in a study by Kamimura et al. (2000) [11], 61% of female college students were evening-types. Moreover, compared to their male counterparts, female students took longer to fall asleep after going to bed, had poorer quality of sleep [21, 22], and had worse overall sleeping conditions [2]. Many studies have reported that evening-type sleep habits were related to uncomfortable psychosomatic symptoms, particularly in young women. Examples include, feelings of depression before the beginning of menstruation [23], irritability during menstruation [24], and menstrual pain [23, 25]. Moreover, evening-type sleep habits were reported to be associated with various stress responses, such as awareness of fatigue [11, 25] and lack of concentration [11, 24]. Thus, the relationship between women’s sleep habits and various physical and psychological responses have been reported; however, the mechanism underlying the relationship between sleep habits and mental and physical health remain unclear and, consequently, further research is needed.

In the present study, we examined the effects of problematic sleep habits in female college students with considerable attention to subjective depression and anxiety. This population was chosen as the target population because many studies have reported that mental health problems, such as depression and anxiety, were significantly more prevalent in women than men [22, 26–28]. Thus, it is necessary to elucidate the process by which the sleep habits of female college students affect their mental health. Therefore, in this study, we examined the influence of female college students’ sleep habits on stress responses (i.e., subjective depression and anxiety) and sought to determine the mechanism underlying the relationship between sleep habits and stress responses.

Although there are several ways of measuring sleep, in the current study, we examined sleep habits by focusing on sleep patterns and sleep irregularity. Preliminary studies have measured sleep habits in terms of “morningness-eveningness.” Kleitman [29] defined “morningness-eveningness” as sleep–wake rhythms, and many later studies adopted the latter term “sleep pattern” [14, 30, 31]. Furthermore, the concept of “morningness-eveningness” contains various other elements (e.g., active time zone, ease of waking up, changeability of daily rhythm). To clarify the effect of sleep habits on stress responses and utilize it for intervention programs to promote student health, it is necessary to extract the important elements from sleep habits. Therefore, in this study, we focused on the aspects of sleep pattern and sleep irregularity. Several studies have recently suggested that irregular sleep, such as lack of sleep on weekdays and oversleep on weekends, has an important influence on health [1, 6, 11, 15, 17, 30, 32]. For example, Honda et al. [17] showed that both an evening-type sleep pattern and sleep irregularity were related to poor psychological health. Moreover, Takeda et al. [32] revealed that irregular sleep had a stronger influence on subjective symptoms than sleep patterns. Behind the link between sleep irregularity and poor mental health is that insufficient or irregular sleep can lead to deterioration of stress tolerance [33]. Sleep deprivation or irregular sleep leads to dysregulation of the hypothalamic-pituitary-adrenocortical axis and increases cortisol secretion at night, resulting in a stress response [34]. Accordingly, sleep irregularities are expected to affect subjective depression and anxiety due to poor stress tolerance. It is evident that sleep irregularity is one of the most important aspects of sleep habits and may affect the stress response.

Lazarus and Folkman's transactional model [35] is a framework adopted to explain the relationship between environmental factors and stress responses [30, 36]. Lazarus and Folkman [35] emphasized the conducting of “cognitive appraisals” to determine the relationships between individuals and their environments. Cognitive appraisal is defined as “the cognitive process of assessing how stressful the interaction between individuals and the environment is” [3] and includes the following: (1) the level of threat associated with the stressor (appraisal of threat), (2) how harmful the threat may be (appraisal of hazard), (3) how aggressively the individual intends to address the threat (appraisal of challenge), and (4) the degree to which the situation can be controlled (controllability). Exposure to environmental demands and pressures can result in stress, but there can be individual and group differences concerning the manner and degree of reaction to such stressors, as well as susceptibility and vulnerability to such events. Lazarus et al. [35] noted that individual differences regarding the responses people exhibit in specific stress situations had a strong influence on individuals’ cognitive processes (i.e., cognitive appraisals concerning interpreting stressors. Also, it has previously been clarified that cognitive appraisal strongly influences the selection of individual coping strategies and the expression of the stress response [37].

At the commencement of research investigating the effect of cognitive appraisal of lifestyle on health outcomes, recent studies [30, 36] adopted the transactional model [26] as a framework and reported that cognitive appraisal had an important effect on mental health. For example, Fukui et al. [30] investigated the effect of cognitive appraisals of daily rhythm on stress reactions in Japanese undergraduate students. Barber et al. [36] revealed the mediation effects of hassle appraise and psychological strain as cognitive appraisals between sleep hygiene and well-being in undergraduate students in the US. While these studies showed that cognitive appraisals played an important role, they also revealed the problem that the methods for measuring cognitive appraisal were different. Therefore, we decided to use the Cognitive Appraisal Rating Scale (CARS), which measures four subscales, commitment, influence, threat, and controllability. Suzuki and Sakano [38] developed the CARS scale and examined the relationship between cognitive appraisal and the stress response in substantial stress situations (task stress, interpersonal stress, physical threat stress), finding that the stress response became significant when the appraisal of threat and commitment (corresponding to the concept of “appraisal of challenge”) of a stressful situation was high. Cognitive appraisal plays an important role in the mental health of individuals. In this study, we adopted the framework of Barber et al. [36] using a transactional model, focused on the roles of the four aspects of cognitive evaluation, and clarified the process of the effect of sleep habits on the stress response.

The relationship between sleep habits and the stress response, and between cognitive appraisal and the stress response have been clarified by previous studies [9–17, 23–25, 36, 38]. However, from the findings of previous research, the degree to which cognitive appraisal affects the relationship between sleep habits and the stress response remains inconsistent and unclear. To obtain data that can be useful for improving the sleep habits and mental health of female college students, further investigation of the effects of sleep habits and cognitive appraisal on the stress response is needed. Based on the model of Barber et al. [36], we predicted that cognitive appraisals of sleep habits would mediate the relationship between poor sleep habits and heightened stress responses in female college students. In particular, we predicted that poor sleep habits would be related to (1) the perception that the individual must improve their lifestyle (commitment), (2) the sleep habits that disturb their lives (threat), and (3) low controllability. Further, we predicted that these cognitive appraisals would exacerbate the stress response (i.e., subjective depression and anxiety).

## Method

### Participants

We recruited 321 Japanese female undergraduate and graduate students from a women’s college in Tokyo as participants in the study. The study questionnaire was administered to 400 students, of whom 324 agreed to participate in the study (response rate = 81.0%). The three respondents aged over 30 years old were presumed to have work experience, and their sleep habits and family composition were considered not representative of college students; therefore, these three students were excluded from the final analysis. Data from the remaining 321 participants were used in this study: 290 (90.3%) were undergraduate students, 17 (5.3%) were graduate students, and 14 (4.4%) did not report their student status. The mean age was 19.72 years (*SD* = 1.73). According to Green (1991) [39], the sample size required for multiple correlations with seven predictors was 106 or greater for medium effect sizes and 111 or greater for partial regression coefficients. A power analysis using SPSS found that 309 respondents were required for linear regression analysis with seven predictors (*p* < .05, statistical power was .95, the effect size was large). Therefore, the sample size of 321 patients in this study was considered to be appropriate.

### Procedure

Data were collected from October to December 2012. After the researchers obtained consent from course instructors, the questionnaire was distributed to the students in classrooms at the end of class. The students were informed that the survey was anonymous and that their participation was voluntary. Completion of the survey questionnaire was considered their consent to participate. Participants gave the completed questionnaire to the researchers in the class or dropped it into a collection box.

The present study was performed in accordance with the Declaration of Helsinki and was approved by the Ethics Committee of Ochanomizu University. All statistical analyses were performed using SPSS 27.

### Measures

#### Sleep habits

Sleep habits were assessed using seven items developed for this study by referencing Ishihara et al. [40] and consisted of two subscales: Sleep Pattern and Sleep Irregularity. Sleep Pattern comprised five items measuring the tendency to engage in evening-type sleep habits and was selected from the Japanese version of the Morningness-Eveningness Questionnaire (MEQ; [41], Japanese version [40]). Since the MEQ contained various elements and there were some items to be excluded (e.g., those containing obsolete language, such as “night watch”) for further investigations [30, 42], we only selected items that were relevant to our aim of measuring sleep patterns (i.e., items that represent the sleep patterns of respondents). The Cronbach’s α score for the internal consistency reliability of the Sleep Pattern subscale was .65. It noted that internal consistency is considered sufficient when the α coefficient is .60 or higher [43]; therefore, the reliability was considered sufficient. Examples of items included, “When you have no commitments the next day, at what time do you go to bed compared to your usual bedtime?” and “One hears about ‘morning’ and ‘evening’ types of people. Which ONE of these types do you consider yourself to be?” Items were responded to with a rating of 1 to 4. Higher scores on the Sleep Pattern subscale reflected a greater tendency to have an evening-type sleep pattern.

Two items were used to measure sleep irregularity, which was specially created for this study: for example, “Do you think you have a regular sleep rhythm on weekdays?” and “Do you think you have a regular sleep rhythm throughout the week?” (a reverse-scored item). The Cronbach’s α score for the internal consistency reliability of the Sleep Irregularity subscale was .82. Participants responded to the items using a four-point scale (1 = “strongly disagree” to 4 = “strongly agree”). Higher scores reflect a greater tendency to engage in irregular sleeping patterns.

#### Cognitive appraisals of sleep habits

Cognitive appraisals of sleep habits were assessed using eight items from the Cognitive Appraisal Rating Scale (CARS) [36]. The CARS includes four subscales that assess commitment (appraisal of the extent to which one actively commits to the situation), appraisal of influence, appraisal of threat, and controllability. The CARS has demonstrated sufficient validity and reliability [36]. To measure cognitive appraisals of sleep habits, items that featured the phrase “this situation” were altered to state “my present sleep habit” or “my present sleep habits” (e.g., “I think my present sleep habit is important to me”). Participants rated each item using a four-point scale (1 = “strongly disagree” to 4 = “strongly agree”). Regarding internal consistency reliability, the Cronbach’s α scores of the CARS subscales were as follows: commitment .78, appraisal of influence .82, appraisal of threat .93, and controllability .56. For each cognitive appraisal component, a higher score reflects a greater tendency to use that type of cognitive appraisal.

#### Stress response

Stress response was measured using two subscales from the Stress Self-Rating Scale (SSRS) [44]: Depression (five items) and Anxiety (five items). Since the participants in this study were mostly healthy college students, we used the SSRS scale developed for college students to measure subjective depression and anxiety. Participants rated each item using a four-point scale (1 = “strongly disagree” to 4 = “strongly agree”). Support for the validity and internal consistency reliability of the SSRS was confirmed in a previous study [45]. The Cronbach’s α scores for the Depression and Anxiety subscales were .88 and .85, respectively. The average scores were calculated and used as the scores for the five items of depression and anxiety. Higher scores indicate a stronger stress response in terms of anxiety and depression.

#### Control variables

In the analysis, it was necessary to control the effects of the confounding variables, such as the amount of stressor and dispositional optimism. Previous research has reported that larger the number of stressor events, the stronger the stress response [30], and the higher the score for dispositional optimism, the weaker the stress response [46, 47]. In this study, it was necessary to examine the effects of sleep habits and cognitive evaluation, so it was essential to control the number of stressors experienced in daily life and the degree of optimism, which have a strong effect on stress responses. To include these variables as control variables, they were measured in the questionnaire. The number of stressor events was measured using the 35-item scale concerning the daily stressors of graduate students included in the SSRS [44]. Following the recommendations of Fukui and Fukui (2009) [30], we removed the scale measuring the degree of distress (0 = no stressor, 1 = “none at all” to 4 = “very much”), retaining only the scale concerning whether the stressor had been experienced (0 = “no,” 1 = “experienced”). To determine the stressor score, the average of the stressor event scores of 35 items was used. Thus, in the present study, higher scores reflected a greater number of stressors experienced. Dispositional optimism was measured using the Japanese version of the Life Orientation Test-Revised (LOT-R) [48], which is a measure of generalized positive and negative outcome expectancies [46].

## Results

To confirm the normality of the obtained data, according to Garson (2012) [49], the skewness and kurtosis of all variables (control factors, sleep habits, cognitive appraisals, and stress responses) were examined, and all the values were within ± 1. Furthermore, when the normal Q-Q plots of all variables were scanned, almost all plots were located on straight lines. Therefore, we justified that the normality of assumption was met. Descriptive statistics, including the means and standard deviations of the variables and the reliability coefficients of the scales, are shown in Table 1.

**Table 1:** Descriptive statistics

	*M*	*SD*	*α*
SSRS-Stressor	.55	.19	–
Optimism (LOT-R)	2.63	.47	.72
Sleep pattern	2.50	.57	.65
Sleep irregularity	2.40	.82	.82
CARS-Commitment	2.58	.73	.78
CARS-Appraisal of influence	3.55	.53	.82
CARS-Appraisal of threat	2.41	.90	.93
CARS-Controllability	2.72	.60	.56
SSRS-Depression	2.16	.85	.88
SSRS-Anxiety	2.13	.77	.85

For stressors, for 35 events, “no” was coded as “0” and “experienced” was coded as “1”. For the stressor's score, the average of 35 event scores was used

The results of the bivariate correlation analysis are shown in Table 2. Evening-type sleep pattern was found to be positively associated with commitment (*r* = .28, *p* < .01) and appraisal of threat (*r* = .27, *p* < .01), but negatively associated with controllability (*r* =  − .30, *p* < .01). Further, sleep irregularity was also found to be significantly associated with higher commitment (*r* = .47, *p* < .01), higher appraisal of threat (*r* = .48, *p* < .01), and lower controllability (*r* =  − .43, *p* < .01). In the correlation analyses, sleep pattern and irregularity were found to have a weak positive association with depression but no associations with an appraisal of influence or anxiety.

**Table 2:** Bivariate correlation matrix for variables

			1		2		3		4		5		6		7		8		9
1	Stressor																		
2	Optimism		− .24**																
3	Sleep pattern		− .10		.05														
4	Sleep Irregularity		.03		− .03		.38**												
5	Commitment		.21**		− .04		.28**		.47**										
6	Appraisal of influence		.07		.03		− .03		.00		.14**								
7	Appraisal of threat		.12*		− .16**		.27**		.48**		.62**		.12*						
8	Controllability		− .08		.18**		− .30**		− .43**		− .26**		.12*		− .31**				
9	Depression		.35**		− .38**		.12*		.13*		.26**		.12*		.30**		− .13*		
10	Anxiety		.31**		− .16**		.08		.07		.25**		.06		.10		− .08		.73**

*p < .05, ^**^p < .01

The control variables were found to be significantly associated with stress responses. Specifically, stressors were positively related to depression (*r* = .35, *p* < .01) and anxiety (*r* = .31, *p* < .01), whereas dispositional optimism was negatively related to depression (*r* =  − .38, *p* < .01) and anxiety (*r* =  − .16, *p* < .01).

We hypothesized that sleep habits would predict stress responses, such as depression and anxiety, through cognitive appraisals. Consequently, four multiple mediation models (two for each sleep habit and two for each stress response) were created using Preacher and Hayes’ (2008) procedure (available through SPSS macro) [50], which also allowed for covariates. This approach, using the bootstrap analysis, enabled us to explore mediating relationships, thereby overcoming the limitations of previous methods (assuming normal distribution, or if it is impossible to test the indirect effect; for a review, see Preacher and Hayes 2008) [50]. Indirect effects were tested using a bootstrapping procedure, which involved running 5000 samples with 95% confidence intervals (CIs).

**Fig. 1:**
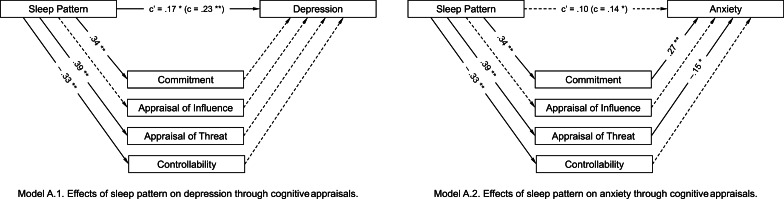
Effects of sleep pattern on stress responses through cognitive appraisals. Analyses control for stressor and optimism. *p < .05, **p < .01.

Table 3 presents the direct and indirect effects of the proposed mediators on the relationship between sleep pattern and stress response. Stressors and optimism were added as covariates. Figure 1 depicts the hypothesized model.

**Table 3:** Results of the multiple mediation model for sleep pattern regarding depression and anxiety

	Depression			Anxiety		
	*b*	*SE*	*t value*	*b*	*SE*	*t value*
*Partial effects of covariates on stress response*						
Stressor	1.19	.24	4.86**	1.04	.24	4.35**
Optimism	− .58	.10	− 5.89**	− .21	.10	− 2.21*
*Effects of sleep pattern on mediators*						
Commitment	.34	.07	5.30**	.34	.07	5.30**
Appraisal of influence	.00	.05	-.10	.00	.05	− .10
Appraisal of threat	.39	.08	4.85**	.39	.08	4.85**
Controllability	− .33	.05	− 6.17**	− .33	.05	− 6.17**
*Direct mediating effects on stress response*						
Commitment	.09	.08	1.12	.27	.08	3.55**
Appraisal of influence	.13	.09	1.48	.06	.08	.70
Appraisal of threat	.11	.06	1.75	− .15	.06	− 2.44*
Controllability	.05	.08	.61	− .01	.08	− .17
*Effects of sleep pattern on stress response*						
Total	.23	.07	3.13**	.14	.07	1.97*
Direct	.17	.08	2.15*	.10	.08	1.31
***Indirect effects***	***b***	***SE***	***95% BCa CI***	***b***	***SE***	***95% BCa CI***
Total	.06	.04	(− .02; .13)	.04	.03	(− .03; .11)
Commitment	.03	.03	(− .02; .08)	.09	.03	(.04; .16)
Appraisal of influence	.00	.01	(− .02; .01)	.00	.00	(− .01; .01)
Appraisal of threat	.04	.03	(− .00; .10)	− .06	.03	(− .12; − .01)
Controllability	− .02	.03	(− .08; .04)	.00	.03	(− .05; .06)
***Model statistics***	*R*	*R*^2^	*F*_(7, 279)_	*R*	*R*^2^	F _(7, 279)_
	.53	.29	15.91**	.40	.16	7.59**

*p < .05, ^**^p < .01

Regarding the relationship between sleep pattern and depression, sleep pattern (a greater tendency to have an evening-type sleep pattern) positively predicted depression (*b* = .23, *t*(283) = 3.13, *p* < .01). Regarding cognitive appraisal, sleep pattern positively predicted commitment (*b* = .34, *t*(283) = 5.30, *p* < .01) and appraisal of threat (*b* = .39, *t*(283) = 4.85, *p* < .01), and negatively predicted controllability (*b* =  − .33, *t*(283) = 6.17, *p* < .01).There was no significant relationship to appraisal of influence. The direct effects of cognitive appraisal types on depression were not significant. The direct effect of sleep pattern on depression was reduced with the inclusion of the mediators and covariates (*b* = .17, *t*(279) = 2.15, *p* < .05). The indirect effect of cognitive appraisals, both cumulatively and individually, showed that cognitive appraisals did not contribute to the prediction of depression (i.e., the 95% CI included zero).

In the results for the multiple mediation model for anxiety, sleep pattern positively predicted anxiety (*b* = .14, *t*(283) = 1.97, *p* < .05). Sleep pattern also positively predicted commitment (*b* = .34, *t*(283) = 5.30, *p* < .01) and appraisal of threat (*b* = .39, *t*(283) = 4.85, *p* < .01), whereas sleep pattern negatively predicted controllability (*b* =  − .33, *t*(283) = 6.17, *p* < .01). There was no significant relationship to appraisal of influence. In addition, commitment and appraisal of threat positively predicted anxiety. The direct effect of sleep pattern on anxiety became non-significant with the inclusion of the mediators (*b* = .10, *t*(279) = 1.31, *p* = .19). Although the total effect was not significant (*b* = .04, 95% CI =  − .03 to .11), commitment (*b* = .09, 95% CI = .04 to .16), and appraisal of threat (*b* =  − .06, 95% CI =  − .12 to − .01) contributed uniquely to the mediation effect (i.e., the 95% CI did not include zero). However, because appraisal of threat did not correlate with anxiety, we could not interpret the indirect effect of threat.

**Fig. 2:**
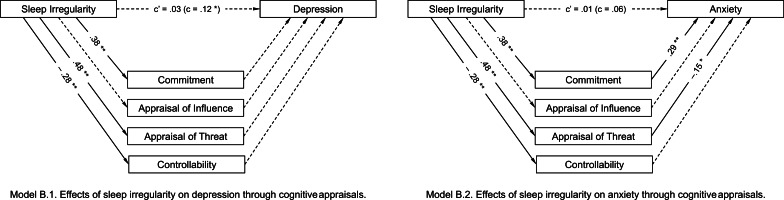
Effects of sleep irregularity on stress responses through cognitive appraisals. Analyses control for stressor and optimism. *p < .05, **p < .01.

Table 4 shows the direct and indirect effects of the proposed mediators in the relationship between sleep irregularity and stress response. Stressors and optimism were added as covariates. Figure 2 depicts the hypothesized model.

**Table 4:** Results of the multiple mediation model for sleep irregularity regarding depression and anxiety

	Depression			Anxiety		
	*b*	*SE*	*t value*	*b*	*SE*	*t value*
*Partial effects of covariates on stress response*						
Stressor	1.07	.24	4.39**	.96	.24	4.07**
Optimism	− .56	.10	− 5.61**	− .20	.10	− 2.06*
*Effects of sleep irregularity on mediators*						
Commitment	.38	.05	8.17**	.38	.05	8.17**
Appraisal of influence	.02	.04	.51	.02	.04	.51
Appraisal of threat	.48	.06	8.58**	.48	.06	8.58**
Controllability	− .28	.04	− 7.30**	− .28	.04	− 7.30**
*Direct mediating effects on stress response*						
Commitment	.10	.08	1.28	.29	.08	3.66**
Appraisal of influence	.13	.09	1.54	.06	.08	.75
Appraisal of threat	.12	.07	1.76	− .15	.06	− 2.26*
Controllability	.00	.08	.03	− .04	.08	− .50
*Effects of sleep irregularity on stress response*						
Total	.12	.05	2.24*	.06	.05	1.10
Direct	.03	.07	.40	.01	.06	.12
***Indirect effects***	***b***	***SE***	***95% BCa CI***	***b***	***SE***	***95% BCa CI***
Total	.10	.04	(.02; .18)	.05	.04	(− .02; .13)
Commitment	.04	.03	(− .02; .10)	.11	.03	(.05; .18)
Appraisal of Influence	.00	.01	(− .01; .02)	.00	.00	(− .01; .01)
Appraisal of threat	.06	.03	(− .00; .12)	− .07	.03	(− .14; − .01)
Controllability	.00	.03	(− .05; .05)	.01	.03	(− .04; .07)
***Model statistics***	*R*	*R*^*2*^	*F*_(7, 279)_	*R*	*R*^*2*^	*F*_(7, 279)_
	.52	.27	14.51**	.39	.15	7.15**

^*^p < .05, ^**^p < .01

Sleep irregularity positively predicted depression (*b* = .12, *t*(283) = 2.24, *p* < .05), commitment (*b* = .38, *t*(283) = 8.17, *p* < .01), and appraisal of threat (*b* = .48, *t*(283) = 8.58, *p* < .01), and negatively predicted controllability (*b* =  − .28, *t*(283) = 7.30, *p* < .01). There was no significant relationship to appraisal of influence. The direct effects of the cognitive appraisal types on depression were not significant, and the direct effect of sleep irregularity on depression became non-significant with the inclusion of the mediators and covariates (*b* = .03, *t*(279) = 0.40, *p* = .69). Analysis of the indirect effect (*b* = .10, 95% CI = .02 to.18) showed that cognitive appraisals as a group contributed to the prediction of depression. However, no individual type of cognitive appraisal showed a unique mediating effect (i.e., the 95% CI included zero).

For the mediation model for sleep irregularity and anxiety, sleep irregularity did not predict anxiety (*b* = .06, *t*(283) = 1.10, *p* = .27). This occurred even though there were significant relationships between sleep irregularity and cognitive appraisals, and between cognitive appraisals and anxiety, besides indirect effects. Since we could not confirm the preconditional relation between sleep irregularity and anxiety, we could not interpret the mediation effects.

## Discussion

In this study, we proposed that based on the model of Barber et al. [34], cognitive appraisals of sleep habits (commitment, appraisal of influence, appraisal of threat, and controllability) would mediate the relationship between sleep habits and stress responses (subjective anxiety and depression). The results of a multiple-mediation-model analysis indicated that cognitive appraisals mediated the relationship between sleep habits and stress responses in a different pattern in the sleep-pattern model and sleep-irregularity model. In the sleep-pattern model, an evening-type sleep pattern was found to be associated with greater depression and anxiety, greater commitment, greater appraisal of threat, and less controllability. Moreover, commitment was associated with greater anxiety, and the mediating effect of commitment was confirmed. This is consistent with past research concerning interpersonal stressors [36]. Only depression was affected by the evening-type sleep pattern. Being awake late at night might directly exacerbate depressive mood. In the sleep-irregularity model, sleep irregularity was found to be associated with greater depression, commitment, appraisal for threat, and less controllability. Meanwhile, no individual type of cognitive appraisal affected depression; however, the cumulative indirect effect of the four types of cognitive appraisal was confirmed. Sleep irregularity was associated with greater commitment, threat, and less controllability, and these cognitive appraisals were related to higher depression. These findings suggest that assessing one's intention to manage evening-type sleep contributes to anxiety, and thinking multifacetedly about the effects of sleep irregularities contributes to depression.

This study builds on past research by exploring the mediating effect of cognitive appraisal on the relationship between sleep habits and the stress response in female college students. Supporting our hypotheses, appraisal of commitment was found to mediate the relationship between sleep pattern and anxiety, and cognitive appraisals, as a whole, mediated the relationship between sleep irregularity and depression, even when controlling for stressors and optimism. Therefore, the relationship between sleep pattern and anxiety and between sleep irregularity and depression can be fully explained by cognitive appraisals, including their significant indirect effects.

These findings have practical implications in that educational institutions can use them when providing sleep-related health education for college students. As afore-mentioned, because the sleep habits of university or college students are becoming irregular and trending toward an evening-type lifestyle, educational institutions need to provide health education for students concerning the dangers of this lifestyle. In general, such health education, which could inform adolescents of the importance of sufficient sleep, might promote students’ awareness regarding their lifestyles. Contrarily, from our research, it was suggested that awareness of commitments, such as “I should go to bed before 22:00” or “I have to fall asleep as soon as possible,” can occasionally have a bad influence on mental health. In other words, when lifestyle change is hindered by existing restrictions (e.g., needing to be awake late at night for a part-time job or to meet friends, or needing to wake up early to attend the class), high awareness of commitment (e.g., I should be living a healthy lifestyle) is likely to increase frustration and anxiety. In such situations, managing or changing how the student cognitively appraises the situation may be an effective way to minimize or alleviate anxiety as a stress response. Therefore, sleep-health education should include a framework of stress appraisals and inform individuals of not only the importance of sleep habits but also the effect of cognitive appraisals. For example, if an individual’s sleep-related lifestyle is difficult to change, it might be more effective for the student to stop searching for a way to improve their sleep habits and avoid trying to drastically change their lifestyle.

In the present study, the participants reported feeling that their sleep habits needed improvement, were threatening to their health and were uncontrollable. In addition, the results indicated that higher commitment to changing one’s sleep habits was related to a stress response, which is consistent with the findings of previous research (cf. Suzuki and Sakano 1998); high commitment was related to high-stress responses in interpersonal relationships (as a stressful situation) [36]. For individuals with evening-type or irregular sleep habits, there is often no clear cause that can be addressed (interpersonal relationship stress as well); therefore, their desire to change but uncertainty regarding the appropriate approach leads to the stress response, and this can harm mental health.

There are several limitations to this study. First, all the participants in this study were female students. In order to generalize the findings of the present study, it is necessary to conduct a survey even for people whose lifestyles are different from those of college students. For example, working people are considered to have a more regular lifestyle than college students. In the case of working people, it is expected to confirm whether similar results can be obtained. Second, the subscale assessing controllability had low internal consistency reliability. Further studies are needed to validate the effect of controllability by using other measures of the constructor evaluating its reliability with other populations. Third, we focused on sleep patterns and sleep irregularities as aspects of sleep habits, so we created the “sleep pattern” scale that utilizes some of the existing scales and the sleep irregularity scale. Since these were the new scales, it may be difficult to simply compare the scores with the previous findings. However, the creation of the new scales has revealed important aspects of health-promoting interventions. In the future, it will be necessary to systematically create a scale that can measure complex factors in sleep. Forth, we measured only subjective sleep and did not collect data on objective sleep. Subjective sleep recognition in healthy individuals, has been suggested to be associated with insomnia symptoms [51]. Thus, the subjective sleep complaint is of interest to clinicians [28] and is an important aspect of empirical research. However, in order to further examine sleep habits, physiological measures must be utilized. Lastly, the possibility that some of the study participants had sleep disorders persists. For example, the prevalence of insomnia has been reported to be 21.4% in Japanese adults [52], and 9.4% in American students [53]. Some of the subjects in this study also have sleep disorders, and it is possible that this affected the results of this study. Therefore, it is necessary to account for such disorders and eliminate those who cannot be labeled as morning- or evening-types.

Despite the above limitations, our results have significance, as we revealed the importance of cognitive appraisals of sleep habits, especially when students have evening-type or irregular lifestyles. Specifically, we observed how people appraise situations or lifestyles that can play an important role not only in stress-coping contexts [35] but also in sleep-habit contexts [36]. Researching cognitive appraisals and sleep habits may lead to the creation of effective health-promoting education for college and university students who are in the process of developing their lifestyles. In the future, to corroborate and disclose the importance of cognitive appraisal of sleep habits, studies targeting people who have low controllability regarding their sleep habits (e.g., night-shift workers) are needed.

## Conclusions

In conclusion, cognitive appraisal of sleep habits, especially appraisal of commitment, may be a pathway through which evening-type and irregular sleep habits affect greater stress responses (depression and anxiety). In particular, increased commitment, a greater sense that one’s lifestyle is threatening to their health, and a lack of controllability may be outcomes of evening-type and irregular sleep habits, which subsequently exacerbate the stress response. In addition, the findings suggest that avoiding thinking about or assessing one’s commitment to improving one’s lifestyle to change their sleep habits has the potential to suppress or alleviate the stress response.

Since our study targeted healthy female college students, we believe that the findings can be used for effective health education, promoting the role of cognitive appraisal. To promote physical health, it is desirable to implement education that propagates the benefits or importance of morningness and sufficient sleep.

However, emphasizing regimented sleep habits can sometimes have harmful effects. Our research revealed that even though it is difficult to improve sleeping habits by oneself, trying to improve habits through certain cognitive appraisals can be stressful. In such cases, avoiding being overly conscious of the need to improve one’s lifestyle is the key to suppressing the stress response. The findings of the present study should be useful for health education related to lifestyle.

## Data Availability

The dataset used and analyzed during the current study is available from the corresponding author on reasonable request.

## Abbreviations

CARS: Cognitive appraisal rating scale
CI: Confidence interval
LOT-R: Life orientation test-revised
MEQ: Morningness-eveningness questionnaire
SSRS: Stress self-rating scale

## References

[1] Takeuchi T, Inugami M, Ishihara K, Fukuda K (2000). Construction of sleep-hygiene scales and classification of sleep patterns in undergraduates. Jpn J Educ Young Child.

[2] Kurokawa T, Ishimura I (2013). Effect of sleep conditions on truancy in university students. Bull Clin Psychol Tokyo Seitoku Univ.

[3] Ohta Y, Saeki K, Ohta K (1994). A study on the lifestyle and the health consciousness of male and female students. Bull Fukuoka Univ Educ Part 5 Art Health Phys Educ Home Econ.

[4] Tokuda K (2013). A preliminary study of life style and mental health in university students: on life style, resilience and sleep. Ritsumeikan J Hum Sci.

[5] MEXT. Enrollment and advancement rate: School Basic Survey; 2013 [cited 2021 April 11]. https://www.mext.go.jp/en/publication/statistics/title01/detail01/1373636.htm#02

[6] Wang F, Bíró É (2021). Determinants of sleep quality in college students: a literature review. Explore (NY).

[7] Honda T, Sakamoto A, Hara E (2006). On the actual state of lifestyle of female university students: an analysis by the intensity level of physical activities on daily life. Fukuoka Jogakuin Univ Bull.

[8] NHK Broadcasting Culture Research Institute. Sleeping time keeps decreasing, male housework time is increasing: from the 2010 NHK Japanese time use survey. 2011. https://www.nhk.or.jp/bunken/english/reports/pdf/report_110401.pdf. Accessed 21 Dec 2018

[9] Hori T. Suimin Shinrigaku. Kyoto: Kitaohji shobo; 2008 (in Japanese).

[10] Harada T, Kadowaki A, Shinomiya H, Takeuchi H (2004). Relationship between watching late night TV and morningness-eveningness of 18–22-year old Japanese students. Sleep Biol Rhythms.

[11] Kamimura Y, Takeda N, Sakuma A, Teraoka C, Kishida N (2000). Effect of night type life style on psychological stress response and dietary habit of women university students. Bull Fac Hum Life Environ Sci Hiroshima Prefect Women's Univ.

[12] Yamaguchi M, Watanabe T, Takagi A, Wakisaka S, Sakane N, Moritani T (2011). Morningness-eveningness preference and the autonomic nervous system activity in the morning among female university students. J Jpn Soc Psychosom Obst Gynecol.

[13] Mecacci L, Rocchetti G (1998). Morning and evening types: Stress-related personality aspects. Pers Individ Diff.

[14] Willis TA, O’Connor DB, Smith L (2005). The influence of morningness-eveningness on anxiety and cardiovascular responses to stress. Physiol Behav.

[15] Kiuchi A, Nakamura T, Arai H, Urai R, Hashimoto K (2010). Relationship between lifestyle and the acquired number of academic credits in first-year college students. Jpn Assoc Univ Phys Educ Sports.

[16] Matsui T, Furumi K, Tsunoda T, Matsumoto K, Teruya K, Tamura H (1989). A study on the health administration of students: The relationship between personal health practices and morning-evening type. J Kyorin Med Soc.

[17] Honda M, Suzuki S, Shirota Y, Kaneko S, Takahashi S (1994). A study on perceived health of morningness-eveningness type and subjective health. Jpn J Health Hum Ecol.

[18] Hamilton N, Nelson C, Stevens N, Kitzman H (2007). Sleep and psychological well-being. Soc Indic Res.

[19] Milojevich HM, Lukowski AF (2016). Sleep and mental health in undergraduate students with generally healthy sleep habits. PLoS ONE.

[20] Dinis J, Bragança M (2018). Quality of sleep and depression in college students: a systematic review. Sleep Sci.

[21] Toscano-Hermoso MD, Arbinaga F, Fernández-Ozcorta EJ, Gómez-Salgado J, Ruiz-Frutos C (2020). Influence of sleeping patterns in health and academic performance among university students. Int J Environ Res Public Health.

[22] Orzech KM, Salafsky DB, Hamilton LA (2011). The state of sleep among college students at a large public university. J Am Coll Health.

[23] Takeuchi H, Oishi T, Harada T (2005). Association between morningness-eveningness preference and mental/physical premenstrual symptoms in Japanese females l2 to 31 years of age. Chronobiol Int.

[24] Nakade M, Takeuchi H, Kurotani M, Harada T (2009). Effects of meal habits and alcohol/cigarette consumption on morningness-eveningness preference and sleep habits by Japanese female students aged 18–29. J Physiol Anthropol.

[25] Negriff S, Dorn LD (2009). Morningness/evenigness and menstrual symptoms in adolescent females. J Psychosom Res.

[26] Suzuki S, Shimada H, Miura M, Katayanagi K, Umano R, Sakano Y (1997). Development of a new psychological stress response scale (SRS-18) and investigation of the reliability and the validity. Jpn J Behav Med.

[27] Zender R, Olshansky E (2009). Women's mental health: depression and anxiety. Nurs Clin North Am.

[28] Regestein Q, Natarajan V, Pavlova M, Kawasaki S, Gleason R, Koff E (2010). Sleep debt and depression in female college students. Psychiatry Res.

[29] Kleitman N (1963). Sleep and wakefulness.

[30] Fukui Y, Fukui T (2009). Cognitive appraisal of sleep habits influences stress reactions among university students. Jpn J Health Psychol.

[31] Roeser K, Meule A, Schwerdtle B, Kübler A, Schlarb AA (2012). Subjective sleep quality exclusively mediates the relationship between morningness-eveningness preference and self-perceived stress response. Chronobiol Int.

[32] Takeda N, Kamimura Y, Teraoka C, Moriwaki H, Sakuma A, Iida T (2001). Relationship between life and diet of women university students by night type life and subjective symptoms. Bull Fac Hum Life Environ Sci Hiroshima Women's Univ.

[33] Lemola S, Schwarz B, Siffert A (2012). Interparental conflict and early adolescents' aggression: is irregular sleep a vulnerability factor?. J Adolesc.

[34] Buckley TM, Schatzberg AF (2005). On the interactions of the hypothalamic-pituitary-adrenal (HPA) axis and sleep: normal HPA axis activity and circadian rhythm, exemplary sleep disorders. J Clin Endocrinol Metab.

[35] Lazarus RS, Folkman S (1984). Stress, appraisal, and coping.

[36] Barber LK, Rupprecht EA, Munz DC (2014). Sleep habits may undermine well-being through the stressor appraisal process. J Happiness Stud.

[37] Folkman S, Lazarus RS, Appley MH, Trumbull R (1986). Cognitive theories of stress and the issue of circularity. Dynamics of stress: physiological, psychological, and social perspectives.

[38] Suzuki S, Sakano Y (1998). Development of a cognitive appraisal rating scale (CARS) and its validation. Waseda Human Science Research.

[39] Green SB (1991). How many subjects does it take to do a regression analysis. Multivar Behav Res.

[40] Ishihara K, Miyashita A, Inugami M, Fukuda K, Yamazaki K, Miyata Y (1986). The results of investigation by the Japanese version of morningness-eveningness questionnaire. Shinrigaku kenkyu [Jpn J Psychol].

[41] Horne JA, Ostberg O (1976). A self-assessment questionnaire to determine morningness-eveningness in human circadian rhythms. Int J Chronobiol.

[42] Honda M, Suzuki S, Ube H, Shirota Y, Kaneko S, Fujima K (1995). Construct validity of the Japanese version of Horne and Ostberg’s morningness-eveningness questionnaire in women college students. Jpn J Health Hum Ecol.

[43] Matsui Y. Shinrigaku ronbun no kakikata. Tokyo: Kawade Shobo Shinsya; 2010 (in Japanese).

[44] Ozeki Y, Haraguchi M, Tsuda A (1994). A covariance structural analysis to the psychological stress process in university students. Jpn J Health Psychol.

[45] Ozeki Y (1993). Refining the stress self-rating scale for university students—toward a transactional analysis. Annu Grad School Comp Stud Int Cult Soc.

[46] Scheier MF, Carver CS, Bridges MW (1994). Distinguishing optimism from neuroticism (and trait anxiety, self-mastery, and self-esteem): a reevaluation of the life orientation test. J Pers Soc Psychol.

[47] Scheier MF, Carver CS (1993). On the power of positive thinking: the benefits of being optimistic. Curr Dir Psychol Sci.

[48] Sakamoto S, Tanaka E (2002). A study of the Japanese version of revised life orientation test. Jpn J Health Psychol.

[49] Garson GD (2012). Testing statistical assumptions.

[50] Preacher KJ, Hayes AF (2008). Asymptotic and resampling strategies for assessing and comparing indirect effects in multiple mediator models. Behav Res Methods.

[51] Ohayon MM, Roth T (2001). What are the contributing factors for insomnia in the general population?. J Psychosom Res.

[52] Kim K, Uchiyama M, Okawa M, Liu X, Ogihara R (2000). An epidemiological study of insomnia among the Japanese general population. Sleep.

[53] Taylor DJ, Gardner CE, Bramoweth AD, Williams JM, Roane BM, Grieser EA (2011). Insomnia and mental health in college students. Behav Sleep Med.

